# A qualitative descriptive study exploring clinicians’ perspectives of the management of older trauma care in rural Australia

**DOI:** 10.1186/s12913-023-09545-x

**Published:** 2023-06-28

**Authors:** Noha Ferrah, Catriona Parker, Joseph Ibrahim, Belinda Gabbe, Peter Cameron

**Affiliations:** 1 School of Public Health and Preventive Medicine, Monash University, Prahan, VIC 3004 Australia; 2 Department of Forensic Medicine, Monash University, The Victorian Institute of Forensic Medicine, Southbank, VIC Australia; 3 Emergency and Trauma Centre, The Alfred Hospital, Melbourne, VIC Australia; 4 Health Data Research UK, Swansea University Medical School, Swansea, Wales

**Keywords:** Older adults, Trauma, Rural, Interview

## Abstract

**Background:**

For older trauma patients who sustain trauma in rural areas, the risk of adverse outcomes associated with advancing age, is compounded by the challenges encountered in rural healthcare such as geographic isolation, lack of resources, and accessibility. Little is known of the experience and challenges faced by rural clinicians who manage trauma in older adults. An understanding of stakeholders’ views is paramount to the effective development and implementation of a trauma system inclusive of rural communities. The aim of this descriptive qualitative study was to explore the perspectives of clinicians who provide care to older trauma patients in rural settings.

**Method:**

We conducted semi-structured interviews of health professionals (medical doctors, nurses, paramedics, and allied health professionals) who provide care to older trauma patients in rural Queensland, Australia. A thematic analysis consisting of both inductive and deductive coding approaches, was used to identify and develop themes from interviews.

**Results:**

Fifteen participants took part in the interviews. Three key themes were identified: enablers of trauma care, barriers, and changes to improve trauma care of older people. The resilience of rural residents, and breadth of experience of rural clinicians were strengths identified by participants. The perceived systemic lack of resources, both material and in the workforce, and fragmentation of the health system across the state were barriers to the provision of trauma care to older rural patients. Some changes proposed by participants included tailored education programs that would be taught in rural centres, a dedicated case coordinator for older trauma patients from rural areas, and a centralised system designed to streamline the management of older trauma patients coming from rural regions.

**Conclusions:**

Rural clinicians are important stakeholders who should be included in discussions on adapting trauma guidelines to the rural setting. In this study, participants formulated pertinent and concrete recommendations that should be weighed against the current evidence, and tested in rural centres.

**Supplementary Information:**

The online version contains supplementary material available at 10.1186/s12913-023-09545-x.

## Background

The challenges faced by government, policy-makers and healthcare providers in designing and managing healthcare systems in response to an ageing population are acutely felt by rural practitioners. Demographic projections estimate a rise in the global population of older adults by 116% equating to 2.1 billion people by 2050 [1]. In Australia, the number of people aged 85 years and over is projected to double by 2042 [2]. While 28% of the general Australian population lives in rural and remote areas, 34% of older Australians (aged 65 and over) live in rural and remote areas [3].

A number of studies have already shown that the number of older adults hospitalised for trauma is growing rapidly in high-income countries [4–6]. In Australia, the number of major trauma patients aged 65 years and older doubled between 2007 and 2016, representing an increase from 25 to 37% of the major trauma population [4]. This demographic trend is at odds with the framework of current trauma systems, which were designed to manage younger patients with major trauma as a result of high-energy mechanisms [5].

Older patients experience greater morbidity than expected for the severity of their injuries, compared to younger patients with equivalent injuries [7–9]. Current protocols dictate that older patients with major trauma are transferred appropriately. Older patients who do not meet the criteria for transfers based on injury severity and mechanism, and haemodynamic instability, are typically managed locally [10]. Therefore, the responsibility of managing older trauma patients, some of whom may be at greater risk of adverse outcomes, rests with rural clinicians [11]. For older trauma patients who sustain trauma in rural areas, the risk of adverse outcomes associated with advancing age is compounded by the challenges encountered in rural healthcare such as geographic isolation, lack of resources, and accessibility [11]. Older trauma patients have a greater likelihood of death than younger trauma patients in a rural environment [7].

The need for specialised trauma care tailored to older adults is increasingly recognised. There are calls from stakeholders in the fields of surgery, nursing, geriatric medicine and trauma research, for the implementation of specialised guidelines and services for older trauma management [12, 13].

New models of care are emerging, including specialised geriatric trauma centres integrating trauma surgery and geriatric medicine, as well as, proactive geriatric consultation services [14–18]. However, it is not certain that these models are adapted to trauma care provision in the rural setting. New care models are designed to target major trauma patients (commonly defined as an Injury Severity Score (ISS) > 12) [19]. Moreover, these are resource-intensive models, requiring specialists in fields such as geriatric medicine, and a critical mass of patients to justify the expenditure and infrastructure for these specialised services [20]. The direct translation of these models into rural areas is problematic due to the scarcity of health professionals, and specialised services such as geriatricians [21]. Thus, specific and novel care models for managing older trauma that are adapted to the rural context are needed. Little is known about the experience and challenges faced by rural clinicians who manage trauma in older adults [22]. An understanding of stakeholders’ views is paramount to the effective development and implementation of a trauma system inclusive of rural communities.

## Aim

The aim of this study was to explore the experiences and perspectives of clinicians who provide care to older trauma patients in rural settings. Specifically, we aimed to describe what factors healthcare professionals in rural areas perceive to enable the delivery of older trauma care; the barriers they encounter, and finally what changes may support their practice.

## Methods

### Study design

We conducted a qualitative descriptive study to understand the experience of health professionals who provide care to older trauma patients in rural Queensland, Australia.

Specifically, we sought to explore the enablers and barriers clinicians encountered, and what changes they want to see to support their practice. The qualitative descriptive study design was chosen as it is suited to obtaining rich descriptions from those experiencing the phenomenon under investigation which was critical to the aims of study [23–25]. In the trauma research literature, the definition of older adults is commonly set at age 60 years and over [26], thus this threshold was also used. With respect to rurality, we used the modified Monash model classification (MMM) of rurality, based on the Australian statistical geography standard – remoteness areas framework [27]. Participants were categorised as primarily practicing in a regional or rural area for MMM categories of three to seven, with three corresponding to towns of 15,000 to 50,000 residents, and seven to very remote communities [27]. Semi-structured interviews were conducted with individual clinicians via videoconference (Zoom© video communications). The study's methods and results are reported according to 'Consolidated Criteria for Reporting Qualitative Research' (COREQ) [28] (See supplementary material, Appendix 1). The study was approved by the Monash University Human Research Ethics Committee (32479).

### Participants

Participants comprised health care professionals (medical doctors, nurses, paramedics, and allied health professionals) with their primary location of practice in rural Queensland, or those practising in a metropolitan setting but providing care to patients transferred from rural areas. In 2019 in Queensland, of 21,735 medical practitioners, 28.4% practised in a rural area, and of 77,258 nurses, 31.5% practised in a rural area [29]. Participants were eligible if they had provided healthcare services within the past 24 months to trauma (defined as physical injury to the body [30]) patients aged 65 years or more.

Purposive sampling was used to attain a breadth of backgrounds and experiences among participants. Recruitment to the study was facilitated by advertisements through research and professional organisations including the Jamieson Trauma Institute, the State wide Trauma Clinical Network, and the Australian Paramedics Association Queensland. Participants were invited to express their interest in taking part in the study by responding to the relevant organisation by email, the primary author (NF) then contacted the participant to confirm eligibility, provide further information on the study, and schedule an interview.

### Data collection

The interview guide was developed based on the relevant contemporary literature on the research topic and the practice knowledge and expertise of the authors. Questions for the semi-structured interview were developed to elicit views of health professionals on their experience of older trauma care in rural settings (Table 1). The questions were structured around three concepts: 1. experience of rural clinicians of a typical case (as defined by the participant) of an older trauma patient; 2. experience of rural clinicians of a memorable case of an older trauma patient and; 3. views of rural clinicians on changes and/or recommendations to improve current practice. The interview guide comprised open-ended questions, and probing questions to elicit more detailed information (Table 1). Participants were asked to recount a typical, and a memorable case of patients they had managed, in order to evoke factors they would subsequently be asked about, including enablers, barriers and recommendations for trauma care.

**Table 1:** Interview guide for semi-structured interviews

***Biography –demographic and professional characteristics***
***Question 1 – A typical case***
* • Can you describe a typical case of a patient over 65 years with trauma that have you cared for?*
* • What things do you remember were done well in that case?*
* • From your perspective, what things could have been done better?*
***Question 2 – A memorable case***
* • I would like you to try and describe a case of an older patient with trauma that stood out for you?*
* • If we take a step back from the two cases you just described, and consider in general, trauma care of older patients in rural areas. What do you think are some of the contributing factors to providing good care?*
* • Again, if we consider trauma care of older patients in rural areas more broadly, what do you think are some of the contributing factors that prevent good care?*
***Question 3—Changes***
* • Tell me about some of the changes you would like to see to support health professionals delivering older trauma care in rural areas?*

Interviews were conducted by the primary author (NF), who tested the interview guide with three pilot interviews. These were also attended by a trained qualitative researcher (CP), for the purpose of mentoring the primary author, and for quality assurance. All subsequent interviews were audio-recorded with participants’ informed consent for verbatim transcription. Informed consent was obtained verbally at the start of each interview, documented, and recorded. Information on the participants’ training and professional background with respect to older trauma and rural healthcare, were obtained at the start of the interview.

### Data analysis

In line with the exploratory nature of the study, and breadth of participants’ experience, thematic analysis was used to identify and develop themes from interviews. All interview transcripts were imported into NVivo 12 (1.6.2), a qualitative data analysis software to support data analysis.

Data analysis was performed independently by two researchers (NF and CP). Themes were discussed for discordance; no further adjudication was required. Data analysis proceeded alongside data collection to enable the themes identified in the earlier interviews to be included in the interview guide and explored in subsequent interviews. Data collection concluded when data saturation was reached, which was defined as when no new information was forthcoming [31]. Both an inductive coding approach using open and axial coding, and a deductive coding approach, were employed to identify key themes in the results. After reading the transcripts, open codes were generated, and the first interviews were inductively re-analysed at the cessation of data collection to ensure no coding was missed in earlier transcripts.

## Results

Of 24 clinicians who expressed their interest in participating in the study, fifteen took part in interviews; 10 medical practitioners, two registered nurses, two occupational therapists, and one paramedic. Eleven participants primarily practiced in a regional or rural area, whilst four participants had their primary location in a metropolitan area; three of whom had a current role in air retrieval of older trauma patients from rural sites. Twelve of 15 clinicians had over ten years of experience since acquiring their professional qualifications (Table 2).

**Table 2:** Demographic characteristics of participants

Profession	Number of participants	Primary location of practice^a^	Level of experience^b^
Medical doctor	10	R = 7; M = 3	S = 9; J = 1
Registered nurse	2	R = 1; M = 1	S = 2
Occupational therapist	2	R = 2	J = 2
Paramedic	1	R = 1	S = 1

^a^*R* regional/rural, *M* metropolitan

^b^Years of experience since participants obtained professional qualifications. J: junior (less than 10 years); Senior (10 years and over)

Several themes were identified within the formulated framework: 1) enablers of trauma care for older people in rural areas; 2) barriers to trauma care of older people in rural areas and; 3) changes to improve trauma care of older people recommended by rural clinicians. In addition,to facilitate guidance for trauma care improvement, the various themes were reported at patient-, clinician- and system-level (Table 3).

**Table 3:** Summary of identified themes and subthemes

	**Factor-level**	**Themes**
Enablers	Patient	*The resilience of people in rural areas*
	Staff	*The experience and training of rural clinicians*
	System	*Relationship between clinicians and the rural community*
		*Delivering better care through a multidisciplinary approach*
		*A robust system for major trauma*
Barriers	Patient	*A life-changing event*
		*Being alone*
		*Not speaking up*
	Staff	*Rural clinicians feel unsupported*
		*Older adult trauma is hard and not sexy*
	System	*The tyranny of distance*
		*The systemic lack of resources in rural practice*
		*A fragmented health system*
Changes	Patient	*Supporting patients in their hospital journey*
		*Enabling discussions on advanced care planning*
	Staff	*Multidisciplinary and coordinated care as standard of care*
		*A coordinator for inpatient and post-acute care*
		*Enabling the training of rural clinicians in older trauma care*
	System	*Improving integration within the health system*
		*Care protocols and standardized referral pathways*
		*Supporting and expanding the use of telehealth*

### Enablers of trauma care

#### The resilience of people in rural areas

Clinicians described patients from rural areas as tough and robust, “*the classic bushy”* (Table 4)*.* Patients were frequently observed to still be working into older age and therefore sustained significant injuries related to farm work, involving machinery, cars, and cattle; *“he got trod on by a big beast” [DR8].* Often, patients sought medical care late, such as one patient who “*[came] in three days after being squashed by a trailer*” [DR2]. Because of perceived stoicism, injuries may be missed or underplayed. Clinicians perceived patients as being attached to their land and community; “*people here really would rather get their care close to home. The people who are living up here, choose to live up here. They don’t like cities.”* [DR2].

**Table 4:** Enablers—Summary of rural clinicians’ experience and example quotes

	**Factor-level**	**Themes**	**Example quotes**
Enablers	Patient	*The resilience of people in rural areas*	*my standard patient is an Italian farmer in his mid-80 s who is still working. The patients under triage their own trauma. They come in three days after being squashed by a trailer. DR3* *he had some fractures in his hands that he didn’t tell anybody about …because he was such a toughie. RN*1 *he was very determined that he was going to get back to the farm. RN1* *even if there aren’t those services set up or available, you’d be able to draw on community support to find someone to help out…those kinds of community connections just exist a bit more in that remote environment. OT2*
	Staff	*The experience and training of rural clinicians*	*These guys have been out there for decades… they’ve seen it all. They can do a lot with nothing. DR6* *I have a couple of orthopaedic surgeons, and two general surgeons [who] are amongst the most physician-like surgeons. They’ve got to be here because you just don’t have back up. You’ve got to be a generalist doctor. DR2* *what the geriatric term was really helpful for, was… learning how to find out about (patients) function … what they’re trying to achieve. Now I’d be comfortable [to]* *have that discussion about goals of care.”* [DR3]
		*Relationship between clinicians and the rural community*	*Clinicians out there would know a lot of the community. That previous knowledge of the patient and their clinical history I think really helps. Whereas you won’t necessarily get that in an urban setting. “They look different” or “They wouldn’t usually present for this”. DR5*
	System	*Delivering better care through a multidisciplinary approach*	*the orthopaedic team was relatively well supported by ortho geris, medical teams. I think that made them less anxious about admitting comorbid people. DR2* *a lot of those trauma patients don’t need to be in a big tertiary hospital, but they need good multidisciplinary, good holistic care. DR5*
		*A robust system for major trauma*	*In every small hospital, we have early notification trauma guidelines where, if someone presents with the usual anatomical, physiological, mechanisms… we would be very quickly looking to move them to the nearest place that can look after them. DR4*

#### The experience and training of rural clinicians

Participants described clinicians as often being the sole practitioner, and thus needed to be generalists with a broad skill set, and the ability to make clinical decisions and provide care with very few diagnostic tools and equipment *“they can do a lot with nothing”* [DR5]*.*

#### Relationship between clinicians and the rural community

Knowledge of patients from previous clinical and non-clinical encounters was also viewed as a strength; *“You know them, you’ve met before, you’ve had a chat and I think that rapport that already exists is protective”* [DR10]*.*

#### A robust system for major trauma

Participants viewed the trauma system as well-rehearsed and oriented towards the management of major trauma cases, with early prehospital notification, protocols with clear identification criteria for critical patients, and fast retrieval to metropolitan trauma centres, “*our system has evolved so that we are much better at the both the pointy end of the resuscitation, access to theatre and the ICU side”* [DR1].

### Barriers to trauma care

#### A life-changing event

Clinicians reported that older rural patients may often live alone and remotely, and lose their independence after sustaining an injury; *“If you’re a 75 year-old who fractured your dominant distal radius and you have no family up there, I don’t know what you’d do.”* [OT2] (Table 5)*.*

**Table 5:** Barriers—Summary of rural clinicians’ experience and example quotes

	**Factor-level**	**Themes**	**Example quotes**
Barriers	Patient	*A life-changing event*	*He had lots of comorbidities, he was a bit of a ticking time bomb. DR1* *the smallest of things can happen and it significantly changes their life pathway. RN2* *For a lot of people in this age bracket, it’s life changing. They don’t get to go back home or when they do, they find it so difficult that they can’t cope. RN1* *If something like this happens, this is life changing and it’s incredibly complex. They often spend significant time in hospital. This is often probably their last 1000 days really. RN1*
		*Being alone*	*In rural areas, people often live quite remote on a farm. They get to a certain age and they have an accident. Then they completely lose their independence from one day to the other because they don’t have the support they need and it’s very difficult to live on a farm by yourself. DR6* *That causes a lot of social dislocation for the patient. They can be quite isolated because their partner may be elderly, can’t visit them – especially with COVID, it’s been really difficult – their families can’t visit them, especially when they’re palliative. [DR4]* *Old people who sustained terrible intracranial injuries who get put on a flight… six* *hours away from their loved ones, only to be then said, “Oh no, this is palliative… The* *outcome is still the same but they’d be surrounded by loved ones when they died, not at* *(city hospital) with no one there.”* [DR6]
		*Not speaking up*	*They’re just a different generation… they are a more vulnerable generation because* *they don’t advocate like they should. And the country folk particularly.”* [RN1] *You’ll get someone who’s been sick for a month, “Oh I just didn’t want to bother you”* *[RN2]* *“Remember that question you asked me eight times and I told you to ask the doctors? They’re here” “I won’t bother them. They look busy” OT1*
	Staff	*Rural clinicians feel unsupported*	*I don’t think there is specific lack of resources for old people. There’s a specific lack of resources for everything. DR6* *More community support would be better … it’s all a bit thin on the ground. DR3* *[we] are always understaffed…always struggling to get a full trauma team together.* *So often it’s a GP and the nurse and that’s it”[DR4]* *[it is] uncomfortable looking after a patient who’s got some blood in their head…* *what do you do if they do go off (deteriorate)? You’re miles away from help” [DR7]* *Very often in this situation, after hours, I’ll be there by myself with a resident. … you’re pretty much on your own. I can think of a situation where one of our guys who has recently left desperately wanted a hand…. But there was literally no other surgeon… for 300 to 400 kms. DR2* *We have sometimes a spinal service – it’s one consultant, so obviously he can’t work all the time. RN1* *We tried to sponsor [rural clinicians] to go for courses. But it was extremely difficult because they’re so understaffed, they can’t take the time off. DR5*
		*Older trauma is hard and not sexy*	*The fracture is the littlest part, but everything else is super difficult around it. DR5* *[Younger trauma patients] are a great trajectory –they just need a little bit of allied health and then off you go. Versus the older person, “it’s just a couple of ribs,, but they’re going to here for four weeks because allied health can’t get them out of the hospital”. OT1* *It’s paradoxical, but when the mechanism of injury is less… rural patients have less access to care, because it’s either not recognised or they don’t have access to the early imaging that they would normally and decision making. [DR3]* *If you’ve got a 15 year-old who’s got multi system trauma, people get excited about that. But if you say you’ve got an 87 year-old who’s on home oxygen and blah, blah blah, it’s a different dynamic. DR7*
	System	*The tyranny of distance*	*People come here by road up to a couple of hours away. West of here there is essentially no hospital for 800 kms. DR3* *I saw people where we were the first hospital they’d landed in and it was about 14 h after their trauma. DR9* *We talk about these people having to be in theatre within 24 h. These folk are lucky if they get to a hospital with a surgeon within 24 h. DR2*
		*The systemic lack of resources in rural practice*	*There’s often delay in getting the patient back into their community because of the lack of allied health, lack of rehab, lack of brain injury [services]. OT2* *They’ll end up being discharged to one of the outlying hospitals and they just sit in a hospital bed and eventually end up in a nursing home. Whereas many of those patients in the city with better care would be getting back home and living independently. DR4*
		*A fragmented health system*	“*this gentleman with chest injuries lay flat on his back for maybe two to three days* *before … (the specialist centre) got back to us about what they wanted for his spinal* *care. I just think that’s unacceptable care*” [RN1] *I only own it when it is on my own soil, and if it’s not on my soil, then it’s not my [problem]. DR1* *Patients … heli-retrievaled in from out of our district, are not actually eligible for rehab within our hospital. So they need to be transferred back to their local hospital or to a tertiary hospital within their own catchment to be able to complete their rehab. DR10*

#### Being alone

When transferred to another hospital, patients were also isolated, away from their community, thus unable to draw on their usual support. Patients may also die after sustaining trauma, away from their community.

#### Not speaking up

Clinicians viewed older patients, and patients from the country as having different expectations from the health system from patients living in urban areas, *“the people up here don’t complain. They will get most horrendous care and they will never complain”* [DR2].

#### Rural clinicians feel unsupported

Participants emphasised a widespread lack of staffing both in rural hospitals and community services, which was not specific to older trauma care. They identified a lack of staff to manage complex and urgent cases. Participants regarded the lack of specialised clinicians as a cause for patients not having timely access to diagnosis or definitive treatment, or being managed in a way not consistent with clinical guidelines *“when you’re in a rural environment, you don’t have everything there. So your care pathways are different” [DR5].* When there is a specialist, they are often the only person on-call and “*the job is enormously wearing for them (consultants).*” [DR2].

Lastly, with the majority of working hours taken by clinical duties, clinicians did not have the time to participate in professional development, research, or quality improvement activities Participants purported this may also contribute to the difficulty in retaining health professionals in the country “*because we’re not training people here, it means we don’t retain people*” [DR2].

#### Older adult trauma is hard and not sexy

Participants reported that mechanisms of injuries were often not as dramatic as in younger patients, which may contribute to under-triage of older trauma patients *“they’ve been on the ground … because the way ambulance triages elderly falls”* [PM1]. Moreover, trauma in older patients could be perceived as comparatively dull “*something a bit more exciting about the younger person with the motorbike accident and the gunshot wounds*” [DR8].

#### The tyranny of distance/ systemic lack of resources

A major barrier raised by participants was that distances from the injury site could be very large *“distance is the biggest issue we have because … that always delays transfer. And that always delays timely treatment” [DR5], as well as* a lack of hospital beds impeding patient flow, and rehabilitation facilities and community services in rural areas.

#### A fragmented health system

Participants reported that the different health services worked in isolation, a lack of streamlined referral pathways, which could contribute to delays of care when awaiting an opinion from specialised centres. Due to lack of integration in the health system, there was no incentive in providing care to a patient out-of-catchment *“they have to be in the hospital for the hospital to take responsibility for that discharge plan” [OT1].* As a result, patients spent an excessive amount of time in acute beds awaiting transfers, solely to be able to access rehabilitation facilities, outpatient and community services.

### Changes to improve trauma care of older people

#### Supporting patients in their hospital journey

When possible, participants thought that an escort for older patients during retrieval would be beneficial, as is already the standard for children and Indigenous Australian patients, *“if you weren’t able to get a lot of history from the patient, having family (present) can really help” [DR8]* (Table 6)*.*

**Table 6:** Changes—Summary of rural clinicians’ experience and example quotes

	**Factor-level**	**Themes**	**Example quotes**
Changes	Patient	*Supporting patients in their hospital journey*	*the indigenous folk in the Northern Territory always were allowed an escort, which I thought was fantastic – certainly for the elderly patients… you need that support. To have somebody flown in with you and supported while you’re in hospital. RN1* *I can’t imagine anything worse than, the last thing you remember you’re in a car accident and then you wake up in this place where, you’re sore and you don’t know what’s going on. The best thing for delirium is something that means something to you, the awareness of something familiar..,. [having] a next of kin readily available, a surrogate decision maker for that person that’s accessible. DR9*
		*Enabling discussions on advanced care planning*	*In our community, we don’t have good discussions about ceiling of care of elderly patients. A lot of people in nursing homes are scared to do that because they think they’ll get no care. I think there’s a lot of confusion around the language of that and it’s often left to junior doctors… to have those conversations. DR10* *A good discussion early about how this might change their life… to have a clear understanding of the pathway that might occur in hospital and also involving families. RN1* *Having a discussion… once you get to the first smaller hospital in conjunction with the treating specialists at the receiving hospitals. That way, informed decisions about trajectories can be made and patient’s wishes or the patient’s proxies can be assessed. DR9* *It takes a long time. It’s time consuming in a time poor environment. But they get to stay at home and they die at home or in their own town. A lot of the rural doctors don’t want to make those decisions because they live there and they’ll see them at the shops or the pub. Having other people come in and help make those decisions is really important. DR4* *Maybe we’ve got to put in place some trigger, when somebody is a trauma and they* *are over a certain age, there’s a prompt to ask*, *Do they have an ARP (acute* *resuscitation plan)? [DR4]*
	Staff	Multidisciplinary and coordinated care as standard of care	*In old people, it’s not so much the injury because the bone gets treated the same way* *in a 90 year-old as it gets in a 40 year-old… So the ideal situation would be to have* *this system where we have a shared treatment or a surgeon geriatrician [DR5]* *You need a generalist… for a small hospital, a good, general physician is invaluable. In a smaller hospital, you have to get on a bit more. Bigger hospitals can sometimes be a lot harder to have relationship building. If you’re a smaller centre, if you’ve got a way of having a joint care model that has a general physician and your surgeon working together, then that’s a good model for those patients.DR2*
		*A coordinator for in-patient and post-acute care*	*They gone from seeing us twice a day, making sure everything is working to now being by themselves, not really sure about the medications they’re meant to take, when they’re meant to come back in. DR7* *Someone who actually telephones them and checks they’re okay would go a long way to making sure that we avoid complications and that they’re either not overdosing themselves on analgesia we’ve given them, or underdosing themselves. DR9* *A trauma nurse navigator post-discharge, would be very valuable in the older person. This is that link between the hospital and the person’s home because once they’re discharged and go back to the GP, they might miss the outpatient clinic because they didn’t have it coordinated for them. DR10* *A nurse practitioner fills that gap between discharge and GP, especially with regards to analgesia, doing chest x-rays, wound care and lots of other psychological support [DR6]*
		*Enabling the training of rural clinicians in older trauma care*	*A stronger emphasis on the types of cases we actually see frequently, regularly rather than the higher acuity cases. It’s this paradox. We train for something we might do once a year, but the elderly faller gets maybe a couple of hours of training [PM1]* *To have your radar up; awareness and greater focus and management of the smaller things, that if not done properly, lead to somebody who might have been leading an independent life, to be nursing home-bound or wheelchair-bound, for injuries that in a younger person wouldn’t necessarily lead to that outcome [DR5]* *Education has to be in the rural hospital because they’re so understaffed, they can’t take the time off… that’s the only thing that’s sustainable. And you need to tailor it to their needs. It doesn’t make sense to do rural trauma education and talk about REBOA. DR5* *We should be going a week every couple of years, through one of the big trauma centres and just doing trauma [education]. The skills are really heavily concentrated down there. DR2*
	System	*Improving integration within the health system*	*It’s better to get them into the right place at the right time, but then you don’t want to overwhelm the system.* H*ow do you put the line for when you’re going to pull the trigger? DR1* *To use each facility to the capacity they can for as long as they can, but recognising early on, if they do need a tertiary level care, bringing them down to the place they* *need. [DR6]* *Accessibility to a universal medical record, would be handy. All you need on it is comorbidities or recent medications and next of kin. That’s just good care, not only patient centric care but telling next of kin that their loved one has been involved in a car accident. DR10* *The problem is we are funded at individual sites and so there is no financial benefit for thinking bigger and broader. So how do you get the administrative side of things to understand the clinical benefit is if we all talk together. DR1*
		Care protocols and standardized referral pathways	*Whether or not we should have different criteria for older people. Do you do it on age? Do you do it on frailty? Other factors? DR1* *However it’s incorporated, it needs to be in a way that is rapidly assessable, by paramedics or pre-hospital clinicians. You’ve got this information overload and you’ve got to add another layer of information. DR7* *A documented pathway of care that emphasises the risk related with geriatric trauma, deciding who goes home, who stays and who goes to [the city]… some guidance on who to image. DR2* *Flowcharts and straightforward evidence-based contemporary, ‘this is what you do in this case’ without it being a bible. DR9* *For the multi trauma patient, it’s an *ad hoc*, phone call to phone call. There’s no point a rural generalist with 20 years’ experience, trying to get advice from one of our junior doctor. DR1* *A system whereby there can be early access to a senior clinician at subspecialty level… for particular injury patterns or severity or important* *decision making, that would go straight to a fellow or consultant level* [DR4] *A trauma lead that you can just ring as a single point of contact for trauma. When you’re not sure what speciality teams need to be involved or you don’t want to do that hour and a half ring around to all the teams who need to do something but none of them want to admit the patient… that could be helpful… DR2*
		Supporting and expanding the use of telehealth	*When it’s set up properly, you can set the cameras… [to see] the monitor, where the vital signs were and the other side, I could look at the patient. It’s like a chameleon [DR9]* *With telehealth, how much of an overreach do you have to support versus guide versus tell. It’s often more of a senior clinician that just needs a bit of a handhold and advice as opposed to direct supervision. DR10*

#### Enabling discussions on advanced care planning

Participants identified advanced care planning as a major area for improvement. The likely trajectory of the patient in light of their injury and current health state should be clearly explained to them, and should ideally involve senior clinicians and be given sufficient time. Additional recommendations put forward by participants included a documented advanced care plan as part of the retrieval checklist.

#### Multidisciplinary and coordinated care as standard of care

Participants advocated for a holistic approach, with early review by a general physician or a geriatrician *“a lot of those trauma patients don’t need to be in a big tertiary hospital, but they need good multidisciplinary, holistic care” [DR6].* Management should be based on an integrated or shared model of care between physicians and surgeons.

#### A coordinator for inpatient and post-acute care

Participants advocated for a dedicated position for care coordination in and out of hospital “*a care coordinator who fights for the patient so that we have one bus that brings them in, they have their whole day of appointments and then go home again” [RN1]*, as well as to provide a link back to the patient’s relatives and primary care physician.

#### Enabling the training of rural clinicians in older trauma care

Participants overwhelmingly advocated for more education on older trauma care. Effective education programs require engagement of participants *“if people don’t understand why they’re doing something… it gives no benefit” [DR1],* should be tailored to rural practice, and ideally be taught in rural centres. However, participants identified the lack of staff cover to take professional leave as a major barrier to accessing education, and proposed this may be remediated through covering rosters within individual professional organizations.

#### Improving integration within the health system

Participants thought that streamlining patient care would be facilitated by integrating the various health services into a trauma system *“a robust system in which we have clearly identified a feeder system… where …there are actual people responsible for the support throughout that network” [DR1].*This would require an accurate understanding of the level of care that can be provided at various locations and the designation of different levels of trauma services with capabilities to manage various aspects of geriatric trauma, e.g. integrated physician/surgeon care model, regional anaesthesia and observation in ICU. This would require governance with *“a state-wide trauma coordinator… who is the first port of call as a senior clinician to assist with decision making” [DR3],* and linking medical records.

#### Care protocols and standardized referral pathways

Participants also recommended a system that would assist in the early identification *“a better trigger at triage that says this person is a risk”*, that is simple and easily applied in triage. Yet triggers should not be oversensitive *“and rushing everybody to the higher priority. You don’t want to be the boy that cries wolf with everybody”* [PM1].

Protocols for common injury patterns (e.g. chest trauma, anticoagulation, head injury) that were easy to follow were thought to be particularly useful in rural settings. Streamlined referral pathways were also highly valued by participants.

#### Supporting and expanding the use of telehealth

Participants identified telehealth as a useful tool. Telehealth was sometimes used in resuscitation bays for the management of trauma patients, and could be expanded to other inpatient settings. For instance, for remote assessment of patients by specialized services in metropolitan centres “*having some sort of administrative agreements, saying that we (can) make decisions based on the information that we see over an electronic platform*” [DR10].

## Discussion

In this study, we gained insight into the experience of clinicians who provide care to older trauma patients in rural settings. To the best of our knowledge, this study is the first to explore the perspective of rural clinicians. Participants identified strengths both amongst older patients and clinicians, such as resilience and resourcefulness, which contributed to good care, despite the systemic lack of resources and integration within the healthcare system. They put forward relevant and practical recommendations, such as a streamlined referral pathway for older trauma patients and a state-wide care coordinator. These recommendations should be assessed against the current evidence, and tested in future studies.

A recurring theme amongst enablers of trauma care was the breadth of experience of rural clinicians, who may be accustomed to practicing in relatively resource-limited environment. Another enabler was the presence of a robust pre-hospital system to manage cases of major trauma. These strengths should be further enhanced in future interventions aiming at optimising trauma care to older patients in rural areas.

The most common barrier was the perceived systemic lack of resources, both material and in the workforce. The dearth of staff in general, and of specialists, and the ensuing strain on rural clinicians, was thought to contribute to sub-optimal care and lack of staff retention of staff. Disproportionate attrition of healthcare workers in rural compared to urban areas has been described in the literature, as were strategies to enhance staff retention [32].

With the exception of pre-hospital care, participants viewed the health system in their state as highly fragmented. The fragmentation of healthcare, and poorer outcomes and inefficiencies that ensue are well known [33], and form the rationale for the creation of trauma systems [34]. Participants felt that the management of older trauma patients from rural areas transcends the boundaries of designated health services. Participants thus proposed that a centralised system of governance and funding, akin to pre-hospital care, should be designed to streamline the management of older trauma patients coming from rural regions. Another recommendation consisted of a case coordinator to facilitate transitions of episodes of care, from in-hospital to outpatient and primary care. An equivalent role in cancer care is thought to be of particular benefit to rural patients [35].

Whilst there is a dearth of studies on the perspectives of rural clinicians on older adult trauma care, one study examined the views of rural clinicians on care of the general adult trauma population. This study also identified that the lack of support for rural clinicians was compounded by the lack of resources, putting extra pressure on healthcare staff [22]. Participants in this study also identified the fragmentation of the healthcare system as a major barrier to providing trauma care [22].

### Implications for trauma systems

Rural clinicians face substantial challenges in providing care to order trauma patients. The perceived lack of support and ensuing attrition is likely to be exacerbated by the growing number of older adults presenting with traumatic injuries to rural centres. This study suggests that possible interventions exist. There is a call from rural clinicians for a centralised system for the management of trauma patients that would integrate rural centres. We should also recognise that some interventions should come from the bottom-up, as they can only be organised locally.

### Future directions

Rural clinicians and older patients are important stakeholders, and should be included in discussions on adapting trauma guidelines in the rural setting. Future studies should assess the validity of these recommendations against the current evidence. For instance, the validity and benefit of a trauma-specific frailty index in aiding rural clinicians in early identification of patients at risk of adverse outcomes, may be tested [36].

The challenges faced by rural older trauma patients have previously been investigated post-discharge [37, 38], but not during their hospital journey. Future studies should examine enablers, barriers and need for change from the perspective of patients during their hospital admission.

### Strengths and limitations

To the best of our knowledge, this is the first study to examine the experiences of rural clinicians involved in the management of older trauma. Using a qualitative approach enabled us to gain in-depth and rich information on several aspects of older trauma care in the rural context. Moreover, the evidence base for trauma care of older adults in rural areas is sparse, in part due to the lower volumes of patients and participants, and the lack of research opportunities [21]. Although, our findings are specific to the local context of the study, they may prompt consideration of rural trauma care provision in countries with equivalent healthcare and trauma systems, such as North America and Scandinavia [22]. There are also important limitations to consider in this study. As participants were identified through voluntary professional organizations, there may be a selection bias. Participants who were made aware of the study, and who agreed to be interviewed may have perspectives that may be different from non-participants who were either not aware of the study or did not agree to participate. The perspective of participants interviewed here may therefore not be generalizable to all clinicians involved in managing older trauma patients in rural areas. Another important limitation is that perspectives gained here were solely from care providers, rather than consumers.

## Conclusions

Rural clinicians are important stakeholders who should be included in discussions on adapting trauma guidelines to the rural setting. In this study, participants formulated pertinent and concrete recommendations that should be weighed against the current evidence, and tested in rural centres.

## Supplementary Information

**Additional file 1. **

## Data Availability

The datasets generated and/or analyzed during the current study are not publicly available as this would compromise the privacy of individual participants who partook in the interviews, but are available from the corresponding author on reasonable request.
